# Acute hydrops followed by corneal perforation five years after corneal cross-linking for keratoconus

**DOI:** 10.5935/0004-2749.20230059

**Published:** 2023

**Authors:** Vesna Jovanovic, Ljubisa Nikolic, Theo G. Seiler, Mirko R. Jankov

**Affiliations:** 1 Faculty of Medical Dentistry, University of Belgrade, Belgrade, Serbia.; 2 Centre for Microsurgery LaserFocus, Belgrade, Serbia.; 3 Department of Ophthalmology, Inselspital, Bern University Hospital, Bern, Switzerland.; 4 Institut für Refraktive und Ophthalmo-Chirurgie, Zurich, Switzerland.

**Keywords:** Corneal injury, Corneal perforation, Keratoconus, Hydrops, Lesão da córnea, Perfuração da córnea, Ceratocone, Hidropsia

## Abstract

We report a case of acute corneal hydrops followed by corneal perforation five years after corneal cross-linking for keratoconus. A healthy 24-year-old female patient underwent Dresden protocol cross-linking in her left eye due to advanced keratoconus. After five years of a stable cornea, she returned with epiphora, blurred vision, and a soft left eye. Acute hydrops and corneal perforation were diagnosed. There was no history of pregnancy, atopy, eye rubbing, trauma, or contact lens use. Local antibiotic and eye patching were applied. Three months after the resolution of the acute episode, she retained useful visual acuity with no need for further surgery. Although cross-linking efficiently halts keratoconus, progression can occur, leading to corneal hydrops and perforation, even in the absence of any risk factors.

## Abstract (pt)

Este é o relato de um caso de hidropisia aguda seguida de perfuração corneana cinco anos após reticulação corneana para ceratocone. Uma paciente saudável de 24 anos foi submetida a reticulação corneana no olho esquerdo pelo protocolo de Dresden, devido a um ceratocone avançado. Após cinco anos com a córnea estável, a paciente retornou com epífora, visão turva e amolecimento do olho esquerdo. Foram diagnosticadas hidropisia aguda e perfuração corneana. A paciente não tinha história de gravidez, atopia, fricção ocular, trauma ou uso de lentes de contato. Foram aplicados um antibiótico local e um tampão oftalmológico. Três meses após a resolução do episódio agudo, ela manteve uma acuidade visual útil, sem necessidade de novas cirurgias. Embora a reticulação interrompa de forma eficiente o ceratocone, pode ocorrer progressão, levando a hidropisia e perfuração da córnea, mesmo na ausência de fatores de risco.

## INTRODUCTION

Corneal hydrops is a rare complication of keratoconus (KC) with an incidence of 1.43 per 1000^(1)^. Clinical manifestations of this condition include marked corneal swelling, ocular irritation, photophobia, and decreased visual acuity (VA). A natural course usually leads to self-healing and scar formation. Very rarely, corneal hydrops is followed by corneal perforation, which is usually associated with vigorous eye rubbing or atopy^(2)^. The introduction of cross-linking (CXL) has changed the course of KC progression with a high rate of success in halting the disease^(3)^. Yet, three reports^(4-6)^ revealed that KC can progress to corneal hydrops months to years after CXL. To our knowledge, this is the first described case of acute hydrops followed by corneal perforation five years after CXL.

## CASE REPORT

A 24-year-old woman with no systemic disease or known allergies presented with bilateral advanced KC (right eye (RE) stage IV and left eye (LE) stage II by Krumeich classification), showing best-corrected visual acuity (BCVA) of counting fingers in the RE and 20/50 in the LE. Corneal tomography (Pentacam/Allegro Oculyzer, WaveLight, Erlangen, Germany) showed maximum corneal curvature of 80.3 D and 52.9 D in RE and LE, respectively. The thinnest pachymetry measured with Pentacam was 375 µm and 481 µm in RE and LE, respectively.

It was decided to proceed with the standard Dresden CXL protocol in LE while RE was scheduled for corneal transplantation. The procedure for LE went uneventful, and six months later the patient’s BCVA was 20/40 in the same eye. The cornea and BCVA remained stable during yearly check-ups.

**Figure 1:**
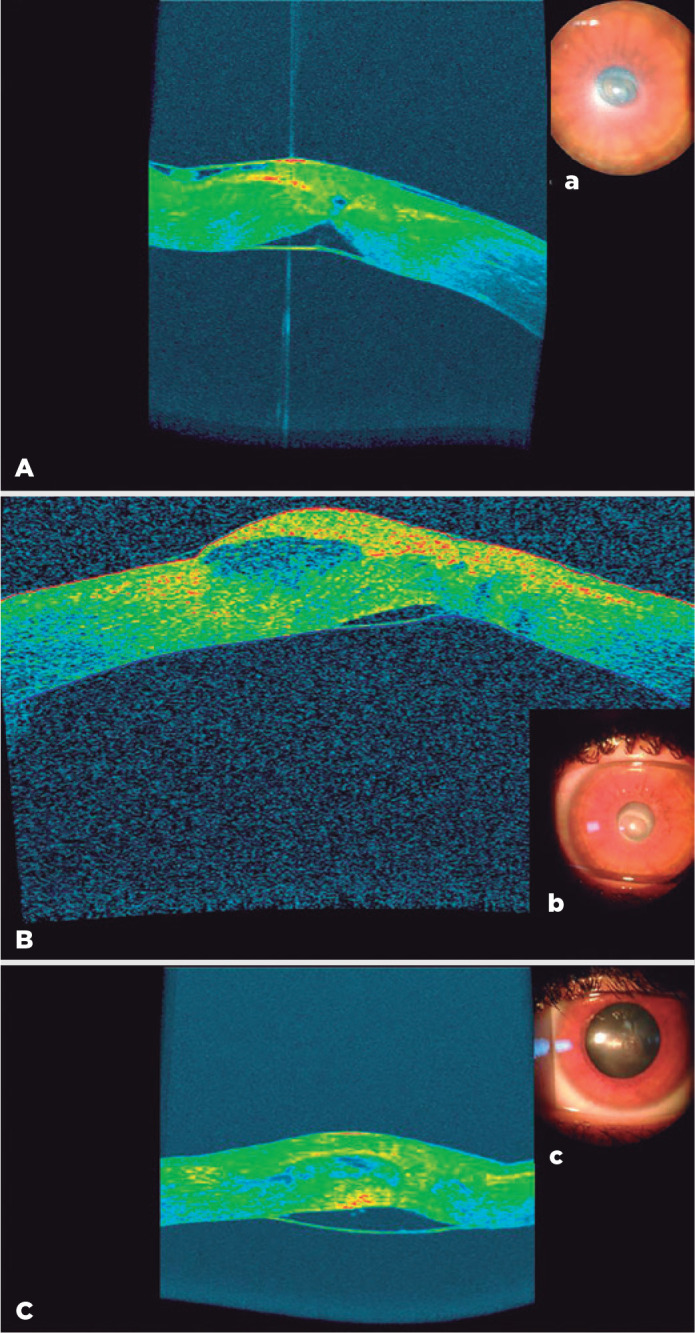
Anterior segment optical coherence tomography image (A) and slit-lamp findings (a) at the onset of acute hydrops, after epithelium healed (B,b), and after resolution of the hydrops (C,c). There is Descemet membrane detachment and fluid-filled space in the stroma and beneath the epithelium.

**Figure 2:**
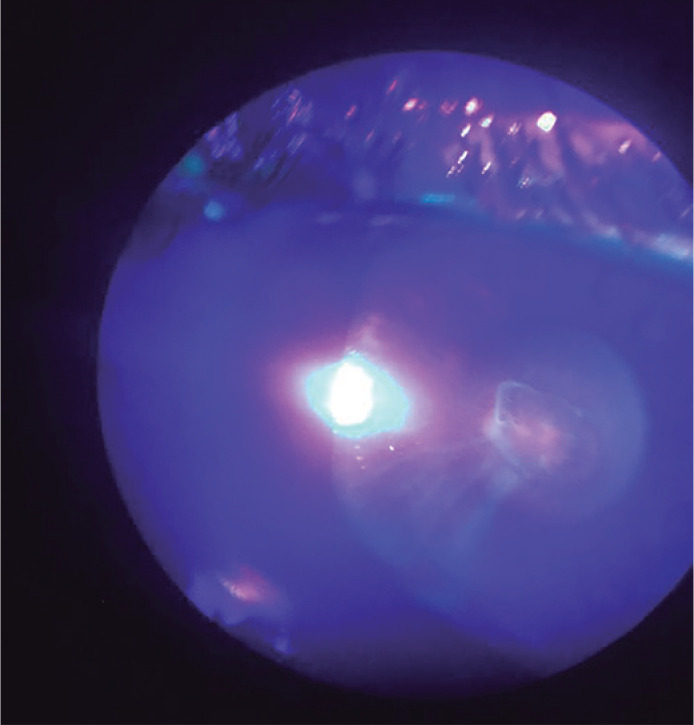
Positive Seidel test (photo taken by a phone attached to the slit-lamp).

Five years after the procedure, and five months after the last check-up, the patient returned complaining of epiphora, photophobia, and reduced vision in her LE. The symptoms lasted for four days, and the patient noticed that the eye was very soft. There was no history of pregnancy, eye rubbing, or any trauma, including contact lens use. On presentation, the uncorrected VA was 20/200 (pinhole 20/50) in LE. Biomicroscopy of LE showed a large centrally located epithelial bulla approximately 2 x 3 mm with very discrete stromal swelling (Figure 1A) and positive Seidel test, defining the diagnosis of an acute corneal hydrops with corneal perforation (Figure 2).

LE tomography showed maximal corneal curvature of 60.2 D. Anterior segment optical coherence tomography (OCT; AS - OCT, SOCT Copernicus Revo, Optopol technology, Zawiercie, Poland) showed Descemet membrane detachment and interlamellar and subepithelial fluid accumulation (Figure 1a). The patient was treated with antibiotic eye drops Floxal (Bausch and Lomb, Rochester, NY, USA) four times daily and a pressure eye patch. Five days after the initial treatment, Seidel test was negative (Figure 1B,b).

By 3 months after the onset of hydrops and corneal perforation, Descemet membrane was partly reattached and corneal edema with epithelial bulla had resolved (Figure 1C,c). Tomography showed corneal scaring with accompanied flattening of the cornea with K_max_ of 52.2 D. In LE, uncorrected VA was 20/200, and BCVA was 20/60. No further surgery was needed.

## DISCUSSION

Acute corneal hydrops is a stressful condition for keratoconic patients as its sudden onset is followed by a dramatic decrease in VA. Even in a pre-CXL era, progression of KC to corneal hydrops was considered a rare complication^(1)^. Rupture of Descemet`s membrane leads to an aqueous humor influx in the corneal stroma and subsequent marked corneal edema, which usually resolves spontaneously within two to four months, resulting in scar formation with consecutive flattening. Very rarely, in severe cases, the epithelium can also break, leading to corneal melting, potentially followed by perforation. The estimated rate of corneal perforation in hydrops is 3.4%^(1)^. Atopic disease and vigorous eye rubbing are considered risk factors for corneal perforation in patients with KC^(2)^.

The introduction of CXL has changed KC treatment, with a high rate of stopping the disease progression^(3)^. The Dresden protocol^(3)^ was proven successful in numerous articles, and five years ago we also used this protocol to treat our patient. Although the progression of the disease can occur after CXL, our patient was stable for five years after the procedure (both tomography and BCVA remained stable during yearly check-ups). Five years after uneventful CXL, corneal hydrops occurred, followed by corneal perforation, without a history of atopy, eye rubbing, or any other trauma.

A literature search in PubMed using the terms corneal collagen cross-linking, corneal cross-linking, CXL, and corneal hydrops found only three case reports.

Antonis et al.^(4)^ reported a case of acute corneal hydrops in a 15-year-old boy three years after standard CXL and intracorneal ring segments implantation. It was suspected that young age, several episodes of allergic conjunctivitis, and eye rubbing were risk factors associated with KC progression after CXL.

Asano et al.^(5)^ reported a case of acute hydrops 3.5 years after epi-on accelerated CXL in a 20-year-old male, and again, eye rubbing was suspected to be a possible causative factor. Insufficient CXL can be assumed due to the used transepithelial protocol.

Stock and al.^(6)^ reported a case of a 26-year-old woman who developed acute hydrops 7 months after uneventful standard CXL in one eye during the fifth month of pregnancy. Hydrops resolved, and the authors postulated that CXL led to the greater resistance of aqueous passage through the cornea, which accelerated the healing process.

In our case, CXL did not prevent aqueous humor from passing through corneal lamellae up to the epithelium, which eventually led to corneal melting and perforation. Even though epithelium healed quickly, edema subsided slowly over three months and Descemet membrane did not attach completely. It seems that CXL did not influence the natural course of hydrops resolution. Recently, a new approach using intracameral gas injection and pre-Descemet sutures has been suggested to accelerate healing response^(7)^, which could have been beneficial in this case. Our patient did not have any of the stated risk factors in the reported cases for developing hydrops or corneal perforation.

In conclusion, we present a case of KC progression five years after standard CXL protocol, which developed acute hydrops and corneal perforation in the absence of any known risk factors. To our knowledge, this is the first described case of acute hydrops followed by corneal perforation five years after CXL. Although CXL is an efficient and safe method in halting the disease, patients and surgeons should be aware of such possible late complications.

## References

[1] Barsam A, Petrushkin H, Brennan N, Bunce C, Xing W, Foot B (2015). Acute corneal hydrops in keratoconus: a national prospective
study of incidence and management. Eye (Lond).

[2] Nivenius E, Montan P (2015). Spontaneous corneal perforation associated with atopic
keratoconjunctivitis: a case series and literature review. Acta Ophthalmol.

[3] Wollensak G, Spoerl E, Seiler T (2003). Riboflavin/ultraviolet-a-induced collagen crosslinking for the
treatment of keratoconus. Am J Ophthalmol.

[4] Antonios R, Dirani A, Fadlallah A, Chelala E, Hamadeh A, Jarade E (2016). Acute corneal hydrops 3 years after Intra-corneal ring segments
and corneal collagen cross-linking. Middle East Afr J Ophthalmol.

[5] Asano S, Miyai T, Toyono T, Aixinjueluo W, Yoshida J, Usui T (2019). Late corneal acute hydrops in ineffective accelerated
transepithelial corneal crosslinking in a patient with
keratoconus. JCRS Online Case Rep.

[6] Stock RA, Thumé T, Bonamigo EL (2017). Acute corneal hydrops during pregnancy with spontaneous
resolution after corneal cross-linking for keratoconus: a case
report. J Med Case Reports.

[7] Yahia Chérif H, Gueudry J, Afriat M, Delcampe A, Attal P, Gross H (2015). Efficacy and safety of pre-Descemet’s membrane sutures for the
management of acute corneal hydrops in keratoconus. Br J Ophthalmol.

